# Emergency Department Boarding of Non-Trauma Patients Adversely Affects Trauma Patient Length of Stay

**DOI:** 10.7759/cureus.10354

**Published:** 2020-09-10

**Authors:** Greg Hymel, John J Leskovan, Zachary Thomas, Joshua Greenbaum, David Ledrick

**Affiliations:** 1 Department of Emergency Medicine, Mercy St. Vincent Medical Center, Toledo, USA; 2 Department of Trauma Surgery, Mercy St. Vincent Medical Center, Toledo, USA

**Keywords:** emergency department, length of stay, crowding, trauma patients, non-trauma patients

## Abstract

Introduction

Emergency Department (ED) boarding delays initiation of time-sensitive protocols for trauma patients and makes them susceptible to increased mortality and morbidity. In this study, we compared the ED boarding times of non-trauma patients and ED length of stay (LOS) of trauma patients.

Methods

This was a single-center retrospective cohort study in a Level 1 trauma center. The median boarding time among non-trauma patients and ED LOS among trauma patients was determined by month between the period of April 2018 to March 2019. Linear regression and Pearson correlation coefficient were used to express the magnitude and direction of the relationship between these two variables.

Results

During the study period, the mean number of non-trauma patients admitted in our ED per month was 1,154 and trauma patients was 89. The mean of the median boarding time per month for non-trauma patients was 76 minutes, and the mean of the median ED LOS per month for trauma patients was 198 minutes. There was a significant positive correlation between boarding time for non-trauma patients and ED LOS for trauma patients (Pearson correlation coefficient: 0.73; p = 0.007).

Conclusion

The long boarding times for non-trauma patients is associated with ED LOS for trauma patients, indicating that the total patient volume in the hospital contributes to the trauma patient's stay in the ED. Thus, ED LOS of trauma patients can be minimized by improving overall ED and hospital flow, including non-trauma patients.

## Introduction

Emergency Department (ED) boarding in which patients are increasingly held in EDs due to a lack of inpatient bed availability is a growing public health problem in the United States and worldwide [1-3]. This delays definitive treatment, particularly for trauma patients, leading to adverse outcomes, such as longer length of stay (LOS) in the hospital, increased costs, and increased mortality compared to patients with shorter ED boarding [4-7]. Factors that contribute to ED boarding include increased volume of critically ill patients in the ED and hospital, ED crowding creating a backlog of patients waiting for open beds, and the lack of available staffed intensive care unit (ICU) beds [5-6, 8]. ED crowding can increase the median boarding time up to 47% and waiting room time up to 78%, delaying treatment for high acuity patients [6]. ED crowding is also associated with delays in the identification and treatment of time-sensitive conditions, such as acute coronary syndrome, stroke, surgical emergencies, and septic shock [9-10].

Consequently, efforts have been made to decrease the boarding time of trauma patients in the ED [5, 11-12]. However, these interventions are focused on expediting the flow of trauma patients from the ED to the ICU and did not involve non-trauma patients in the ED. It is not clear whether improving overall patient flow will improve the trauma LOS specifically. In this study, we sought to compare the boarding times of non-trauma patients and the ED LOS of trauma patients, hypothesizing that longer boarding times for non-trauma patients prolong ED LOS for trauma patients.

## Materials and methods

This was a single-center retrospective cohort study at Mercy St. Vincent Medical Center in Toledo, OH, which is a Level 1 trauma center with an annual volume of 68,000 visits per year. All patients admitted to the ED, including all trauma patients (trauma alerts, trauma priorities, and trauma consults) visiting the trauma bay, between April 2018 and March 2019 were included in this study. The cases discharged from the ED or transferred to another institution were excluded. The median boarding times (among non-trauma patients) and ED LOS (among trauma patients) were calculated by month during the study period. These data were collected after the most recent ED renovation in which the trauma bays were separated from the rest of the ED to form a dedicated trauma unit designed to alleviate LOS for trauma patients. Such a design allows trauma patients with an alert (patients requiring immediate intervention) and priority status (patients with a potential need for immediate intervention) to not only receive a priority in nursing staff, trauma physicians, anesthesiologists, respiratory therapists, and physicians from outside the ED but also receive an immediate response from all ancillary services, including holding the computed tomography (CT) scanner open until the trauma patient has been imaged. When the time from patient admission to the time of arrival on the floor exceeded 30 minutes, it was defined as boarding, and boarding time (minutes) was used as a surrogate marker to estimate ED crowding. Linear regression and Pearson correlation coefficient were used to express the magnitude and direction of the relationship between ED boarding time (among non-trauma patients) and ED LOS (among trauma patients). The Statistical Analysis System (SAS), version 9.4 (SAS Institute Inc., Cary, NC) was used for the statistical analysis.

## Results

**Figure 1:**
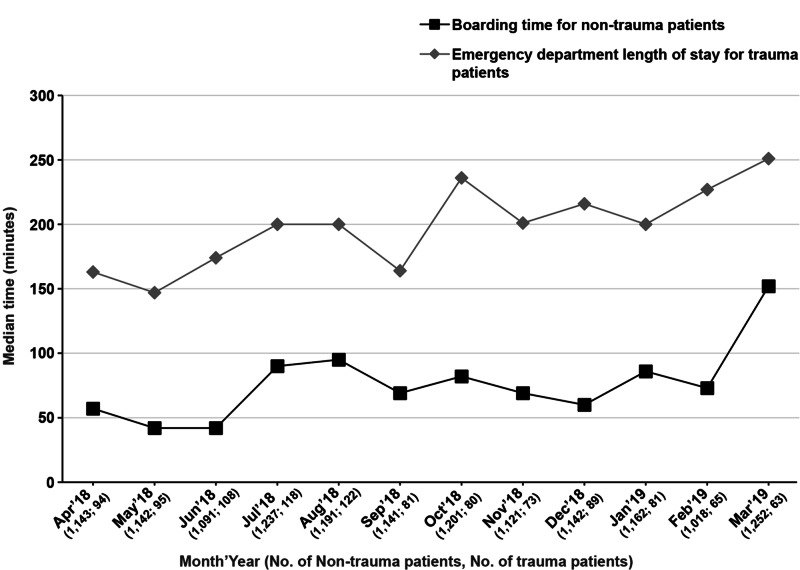
Trends in median boarding time for non-trauma patients and ED LOS for trauma patients by month (minutes) ED: emergency department; LOS: length of stay

**Figure 2:**
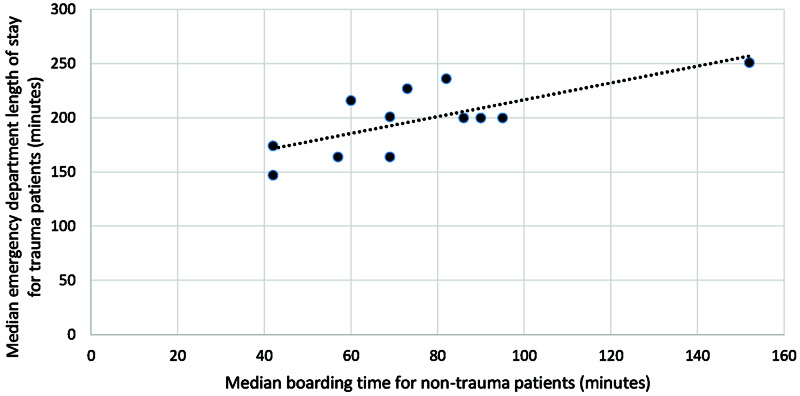
Correlation between median boarding time for non-trauma patients and ED LOS for trauma patients by month (minutes) ED: emergency department; LOS: length of stay

Between April 2018 and March 2019, the mean number of non-trauma patients admitted to the ED per month was 1,154, and for trauma patients, it was 89. The mean of the median boarding time per month for non-trauma patients was 76 minutes (mean IQR: 59 - 88), and the mean of the median ED LOS per month for trauma patients was 198 minutes (mean interquartile range (IQR): 169 - 222) (Table 1). We observed a similar trend in the median boarding time among non-trauma patients and the ED LOS among trauma patients, as indicated by a mimicking pattern of increases and decreases in the median times by month (Table 1, Figure 1). There was a significant and positive correlation between the ED boarding time of non-trauma patients and the trauma patients' ED LOS (Pearson correlation coefficient 0.73; p = 0.007). The linear regression line confirmed this relationship (Figure 2). Based on this data, for every 10-minute increase in the median boarding time in non-trauma patients, the median ED LOS for trauma patients increased by 7.8 minutes.

**Table 1:** Median Boarding Time in Non-Trauma Patients and ED LOS for Trauma Patients by Month (Minutes) § Median time (minutes) ED: emergency department; IQR: interquartile range; LOS: length of stay

Month	Number of Non-Trauma Patients	Number of Trauma Patients	Boarding Time for Non-Trauma Patients ^§^	IQR	ED LOS for Trauma^§^	IQR
Apr 2018	1,143	94	57	21–135	163	97–261
May 2018	1,142	95	42	14–93	147	92–232
Jun 2018	1,091	108	42	13–96	174	125–242
Jul 2018	1,237	118	90	29–209	200	132–355
Aug 2018	1,191	122	95	35–256	200	128–301
Sep 2018	1,144	81	69	23–171	164	111–254
Oct 2018	1,201	80	82	29–194	236	157–355
Nov 2018	1,121	73	69	26–161	201	124–273
Dec 2018	1,142	89	60	24–151	216	138–318
Jan 2019	1,162	81	86	27–204	200	139–295
Feb 2019	1,018	65	73	27–181	227	120–349
Mar 2019	1,252	63	152	45–416	251	157–422
Mean	1,154	89	76	59–88	198	169–222

## Discussion

In this study, we analyzed the ED boarding times for non-trauma patients and ED LOS for trauma patients and identified a positive correlation, indicating that as ED boarding time for non-trauma patients increases, ED LOS for trauma patients also increases, despite institutional efforts to isolate and prioritize the trauma service. Our results suggest a dependent relationship between community-based trauma programs and ED services provided to non-trauma patients; therefore, strategies to reduce ED LOS for trauma cases must account for non-trauma patients in the ED.

Prior studies suggest that increased ED boarding is associated with adverse patient outcomes in different patient populations. ED boarding times greater than two hours is associated with a significant increase in the mortality and hospital LOS of not only trauma patients but also of critically ill and ICU patients [1, 4, 13-15]. For intubated trauma patients, each hour in the ED was associated with an increased risk of developing pneumonia by 20%, resulting in a longer duration of ventilation, prolonged ICU stays, and increased healthcare costs [16]. Furthermore, ED overcrowding was related to 5% greater odds of inpatient death, 0.8% longer hospital LOS, and 1% increased costs per admission [17].

Consequently, efforts have been made at other institutions to decrease the boarding of trauma patients in the ED. Stankiewicz et al. reduced the time to the admission of trauma patients to the surgical ICU from an average of 408 minutes to 143 minutes with the simple expedient of having the surgical ICU nurse coordinate the admission and coming to the ED directly, thus eliminating most of the factors associated with the ED to ICU transition [5]. With this approach, ED nurses saved an average of 265 minutes per patient, reducing the total nursing hours by 146 over 33 patients. Fuentes et al. instituted a protocol to take patients directly from the CT scanner to the ICU, thus reducing ED LOS by 229 minutes [11]. Huang et al. demonstrated a decreased ED LOS for trauma patients by improving communication between the ED and the trauma team [12]. These interventions involved moving trauma patients through the ED without considering the whole ED as a unit. As a result of the findings of this study, we have decreased the number of handoffs and have improved communication by assigning a nurse from the trauma ICU to respond to trauma alerts. This keeps one nurse in charge of the patient throughout the initial phase of care rather than having the nurses in the ED report to the trauma ICU. Such an approach has the potential to not only decrease medical errors through miscommunication but also eliminate the time spent in coordinating the schedule of two nurses to do a patient handoff.

Several factors affect LOS in ED patients, including organizational factors (such as shortage of beds leading to hospital transfer, radiological imaging, or sequential specialist consultations), as well as patient and hospital-specific factors (such as patient age, hospital teaching status, hospital size, and delayed ED throughput of trauma patients) [18-19]. Additionally, Salehi et al. demonstrated that patients under isolation, under telemetry, older patients, and patients with a greater comorbidity burden had prolonged waits in the ED (either as prolonged ED LOS or prolonged boarding) and these prolonged boarding times were associated with greater inpatient LOS [20]. Importantly, ED boarding contributes to the increased morbidity and mortality of trauma patients by delaying the initiation of trauma ICU (TICU)-specific care protocols, which drive the trauma care outcomes, especially during initial resuscitation efforts [4]. Moreover, nursing ratios are lower in the TICU than the ED, which allows for a better implementation of trauma care protocols, such as mechanically ventilated patient care. Unlike ICUs, care in the ED can be fragmented due to interruptions by other patients placing demands on nursing and ancillary personnel's time, as well as the inability to be fully controlled by the physician. At our institution, we have observed an association between the average LOS of inpatients and the amount of boarding time in the ED. We have made every effort to isolate the trauma patients from this institutional chaos. However, ED LOS of trauma patients remains associated with ED boarding of non-trauma patients, which highlights the potential contribution of total hospital volume towards the ED LOS of trauma patients. 

Our findings suggest that the flow of both trauma patients, as well as the flow of the non-trauma patients, through the ED should be improved to minimize the ED LOS of trauma patients. We propose that emergent procedures could take precedence over non-emergent orthopedic procedures, such as sedation and reductions, and radiology studies, such as facial bones or reconstructed spinal scans not relevant to the immediate care of ED patients. Similarly, holding the patient for evaluation by interventional radiology or neurosurgery to go to the radiology suite or operating room prior to going to the trauma ICU could be avoided, despite the convenience of obtaining all the studies in the ED at one time. In fact, decreasing the number of non-emergent scans helped to improve the patient flow in the ED (Perotte R, Lewin GO, Tambe U, et al.: Improving emergency department flow: reducing turnaround time for emergent CT scans. Presented at the American Medical Informatics Assn. Ann. Symp., San Francisco, CA, Nov. 3-7, 2018. www.ncbi.nlm.nih.gov/pmc/articles/PMC6371246/). Additional strategies that improved overall patient flow involved doctor-led triage, rapid assessment, and availability of point-of-care testing in the ED [21]. Thus, both the ED and hospital are interconnected, such that overall patient flow affects the flow of trauma patient through the ED.

Limitations

Limitations of this study include its retrospective nature, short time-frame, and single-center design. Additionally, due to the retrospective design, only an association between ED LOS and boarding for trauma and non-trauma patients can be implied. Importantly, trauma cases are given priority for imaging, testing, and treatment, while consuming available nursing resources, removing them from non-trauma ED patients; therefore, it is plausible to suggest that trauma cases increase boarding times of non-trauma patients. This study was also limited by the fact it was retrospective and certain information could not be reliably abstracted. For instance, it was not clear how many patients with prolonged ED LOS required procedures, such as wound repair, fracture reduction, or further advanced imaging. Lastly, this study does not directly address the effect of ED patient volume for non-trauma and trauma cases on ED boarding and LOS. Larger multicenter studies will be needed to generalize the findings of this study.

## Conclusions

In conclusion, we demonstrate that longer boarding time for non-trauma patients is associated with prolonged ED LOS for trauma patients, highlighting the interconnectedness of the ED and hospital. Thus, to shorten the ED boarding, instead of focusing solely on the trauma process, trauma physicians and administrators should consider ways in which the trauma service interacts with the rest of the hospital system, as the care of a trauma patient requires a multidisciplinary approach.
